# Pouch cancer in familial adenomatous polyposis. Incidence, risk factors and literature review: a propos of three rare cases

**DOI:** 10.1590/0102-67202025000059e1928

**Published:** 2026-04-10

**Authors:** Fábio Guilherme CAMPOS, Carlos Augusto Real MARTINEZ, Renata Nobre MOURA, Adriana Vaz SAFATLE-RIBEIRO, Carlos Frederico Sparapan MARQUES, Ulysses RIBEIRO, Paulo HERMAN

**Affiliations:** 1 Universidade de São Paulo, Faculty of Medicine, Gastroenterology Department, Colorectal Surgery Division – São Paulo (SP), Brazil.; 2 Universidade São Francisco, Postgraduate Program in Health Sciences – Bragança Paulista (SP), Brazil.; 3 Universidade de São Paulo, Faculty of Medicine, São Paulo Cancer Institute, Digestive Endoscopy Unit – São Paulo (SP), Brazil.; 4 Universidade de São Paulo, Faculty of Medicine, Department of Gastroenterology – São Paulo (SP), Brazil.

**Keywords:** Adenomatous polyposis coli, Proctocolectomy, restorative, Colonic pouches, Neoplasms, Adenomatous polyps, Polipose adenomatosa do colo, Proctocolectomia restauradora, Bolsas cólicas, Neoplasias, Pólipos adenomatosos

## Abstract

**Background:**

Development of pouch cancer is a great challenge to both surgeons and patients with familial adenomatous polyposis (FAP) after restorative proctocolectomy (RPC).

**Aims:**

We aimed to present our experience with pouch cancer diagnosis and review literature data regarding incidence and associated risk factors.

**Methods:**

This retrospective study enrolled FAP patients undergoing RPC between 1981 and 2023 in our academic institution. It included only J-pouch stapled patients with at least three years of follow-up. Patients’ demographics and disease features were retrieved.

**Results:**

After excluding seven patients, we selected 87 RPC, and three cases (3.4%) of pouch cancer were identified. They were diagnosed in three men aged 23–40 years at RPC and 41–62 years at cancer diagnosis. Interval from RPC to pouch cancer diagnosis varied from 11.6 to 20 years (average 14.6 years). All patients had colorectal cancers (CRC) detected in the specimen from the index surgery, two of them with multicenter lesions. A brief review of the literature series showed that pouch cancer has been detected in incidences ranging from 0.8 to 3.4%. Male sex, CRC in the RPC specimen, pouch phenotype during follow-up and an association with duodenal adenomas are considered risk factors.

**Conclusions:**

Pouch cancer is a rare event associated with specific risk factors. After RPC, all patients should undergo endoscopic surveillance, with special attention to those who develop an aggressive phenotype during the first decade of follow-up.

## ARTICLE HIGHLIGHTS

- Familial adenomatous polyposis (FAP) is an autosomal dominant disease associated with mutations in the APC gene.
- Cancer prevention is most usually accomplished through restorative procedures such as total colectomy with ileorectal anastomosis (IRA) or a restorative proctocolectomy with ileoanal anastomosis (RPC).
- The development of ileoanal pouch cancer is not so common in patients with FAP even in specialized centers.
- Pouch cancer is a rare disease diagnosed in incidences varying from 0.8 to 3.4% in worldwide FAP series.

## Summary

**CENTRAL MESSAGE:**

Familial adenomatous polyposis (FAP) is an autosomal dominant disease associated with mutations in the APC gene. As a dominantly inherited cancer-predisposing syndrome, the main challenge of FAP management is the significant risk of CRC that requires prophylactic colectomy in a timely manner aiming to reduce colorectal cancer (CRC) risk while maintaining quality of life. Cancer prevention is most usually accomplished through restorative procedures such as total colectomy with ileorectal anastomosis (IRA) or a restorative proctocolectomy with ileoanal anastomosis (RPC). The development of ileoanal pouch cancer is not so common in patients with FAP, even in specialized centers.

**PERSPECTIVES:**

Pouch cancer is a rare disease diagnosed in incidences varying from 0.8 to 3.4% in worldwide FAP series. Male patients, presence of CRC in the RPC specimen, colorectal phenotype, and association with duodenal adenomas are considered the main risk factors. Pouch adenomas develop after both hand-sewn or stapled anastomosis. Pouch polypectomy might prevent the development of adenocarcinomas, as patients under surveillance are diagnosed with more localized diseases.

## VISUAL ABSTRACT

**Figure:**
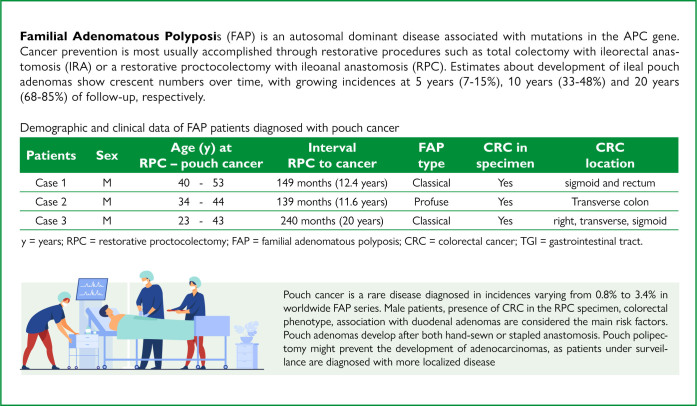

## RESUMO

**Racional:**

O desenvolvimento de câncer na bolsa ileal constitui um grande desafio para o cirurgião e pacientes com Polipose Adenomatosa Familiar (PAF) após a proctocolectomia (RPC).

**Objetivos:**

Apresentar nossa experiência com o diagnóstico de câncer na bolsa ileal e rever a literatura relacionada à incidência e fatores de risco associados.

**Métodos:**

O presente estudo retrospectivo envolveu pacientes PAF submetidos a RPC entre 1981 e 2023 em nossa instituição acadêmica. Incluíram-se apenas doentes com 3 anos de seguimento, Dados demográficos e características da doença foram extraídos dos prontuários.

**Resultados:**

Após excluir 7 doentes, foram selecionados 87 RPC e 3 casos (3.4%) de câncer na bolsa ileal foram identificados. Os tumores foram diagnosticados em 3 homens com 23-40 anos na RPC e 41-62 anos no diagnóstico de câncer. O intervalo entre RPC e câncer na bolsa variou de 11.6 a 20 anos (média 14.6 anos). Todos os doentes tiveram câncer colorretal (CRC) detectados néco espime da cirurgia primária, dois deles com lesões multicêntrica. Uma breve revisão das séris da literatura demonstrou que o câncer de bolsa ileal tem sido detectado em incidências variando de 0.8 a 3.4%. Sexo masculino, CRC no espécime da RPC, fenótipo da bolsa durante o seguimento e associação com adenomas duodenais são considerados fatores de risco.

**Conclusões:**

Câncer na bolsa ileal é um evento raro associado com fatores de risco específicos. Após proctocolectomia, todos os doentes devem fazer vigilância endoscópica, com especial atenção para aqueles que desenvolvem fenótipo agressivo na bolsa durante os primeiros dez anos de seguimento.

## INTRODUCTION

Familial adenomatous polyposis (FAP) is an autosomal dominant disease associated with mutations in the APC gene. While the classic form is associated with polyp formation after the second decade of life and an enormous risk of cancer if left untreated, the attenuated variant expresses a milder phenotype in which polyps and cancer may develop later in life^42^. As a dominantly inherited cancer-predisposing syndrome, the main challenge of FAP management is the significant risk of colorectal cancer (CRC) that requires prophylactic colectomy in a timely manner aiming to reduce CRC risk while maintaining quality of life^53^.

Cancer prevention is most usually accomplished through restorative procedures such as total colectomy with ileorectal anastomosis (IRA) or a restorative proctocolectomy with ileoanal anastomosis (RPC)^9,10,13^. RPC is usually recommended in cases exhibiting profuse colonic polyposis, a high polyp burden in the rectum or those with specific mutations leading to severe disease^12^. It is preferred because it provides a better function^19^.

The technique’s description in 1978^35^ created a false sense of security, as it fostered the belief that it could be possible to minimize the risk of malignancy in FAP. However, pouch polyps may develop either after a stapled or handsewn anastomosis. The first description reported ileal Kock pouch adenomas in 1982^17^, and pouch polyposis was later described in 1987^32^. Different from end ileostomy, ileal pouch-anal anastomosis (IPAA) creates an environment with fecal stasis, villous atrophy, and colonic metaplasia^15^. Thus, the development of pouch adenomas is not a surprising event in patients with underlying genetic predisposition to neoplasia (adenoma, dysplasia, or cancer), either in the ileal tissue of the pouch body or in the remnant rectum or rectal cuff^25,28,29^.

Adenomas may arise in the residual anal transition zone (ATZ) or rectal mucosa deliberately left behind after a stapled anastomosis or even after a handsewn anastomosis due to remnants of rectal mucosa in this area^24^. The so-called “rectal cuff” is a strip of rectum above the dentate line where inflammation and neoplasia may develop^11,12^. Data from the Heidelberg Polyposis Registry and other important studies suggested that, despite mucosectomy, residual rectal mucosa islands or microscopic rectal columnar epithelium may be left behind and become hidden to visual examination during endoscopic surveillance in around 20% of patients, giving support to the idea that mucosectomy down to the dentate line does not eliminate the risk of neoplasia development. Otherwise, confection of a stapled anastomosis also leaves a cuff of residual rectum that houses the stapler cartridge, where adenomas may also develop more frequently when compared to a handsewn anastomosis^16,18,24,27^.

Usually, these low adenomas start to appear about two years after RPC, and incidence increases with pouch age, making surveillance strategies necessary to prevent cancer, despite its rare occurrence. In contrast, pouch body adenomas tend to appear later. A stapled anastomosis is associated with a greater risk^16,27,51^.

Association with male sex, age higher than 18 years at RPC, high adenoma count at index colectomy, advanced duodenal polyposis, gastric adenomas, genotype (APC gene codons 1309 and 1328), increased duration of follow-up, and stapled anastomosis are considered predictive factors associated with pouch dysplasia^7,17,23,27,28,29,39,52^. Nevertheless, some are still highly controversial, such as genotype and colonic adenoma burden^18^. One study found that no patients exhibiting less than 200 colonic adenomas developed pouch adenomas, while 46% of those with more than one thousand polyps did^49^.

The literature regarding neoplastic outcomes within the pouch has displayed inconsistent results^38^. Estimates about the development of ileal pouch adenomas show crescent numbers over time, with growing incidences at five years (7–15%), ten years (33–48%), and 20 years (68–85%) of follow-up, respectively^1,4,8,16,47^.

They tend to be sessile and exhibit indistinct borders with the surrounding ileal mucosa, making assessments of incidence a challenge. The progression of pouch adenomas is slow and, although adenoma incidence seems to stabilize, it is reasonable to affirm that the severity of the neoplasia gets worse with time^23,27,28,49^. In a large study including 212 patients from the Netherlands, the cumulative risk of advanced pathology and cancer were 11.7 and 1%, respectively^11^.

Fortunately, development of ileoanal pouch cancer is not so common in patients with FAP even in specialized centers. The first case of pouch cancer was described by Hoehner et al. in 1994^21^. Since then, less than 50 cases have been reported in literature reviews after primary IPAA^18^.

The development of pouch cancer is a very rare event that poses a great challenge to both surgeon and patient. The present retrospective study was performed to review pertinent data concerning pouch cancer incidence in FAP patients, with the aim of drawing attention to this issue and identifying predictive risk factors. Finally, the analysis of our long-term surveillance data may help to improve our own clinical outcomes.

## METHODS

In the present retrospective study of FAP patients, we refined selection by only enrolling patients who underwent restorative proctocolectomy between 1981 and 2023 and were followed at our academic institution. We included only those with J-pouch cancers diagnosed after a minimum of one previous endoscopic examination. The exclusion criteria were: patients with less than three years of follow-up, those with other forms of pouch, and patients with pouch failure. Since 2007, the patients have undergone surveillance every 1–2 years after primary RPC.

This study was approved by the Department’s Ethical Committee Board. Clinicopathological, endoscopic and management data were extracted from medical charts, and carefully analyzed, aiming to identify potential risk factors involved in the development of cancer in the reservoir. We retrieved patients’ demographics (sex, age at index surgery and at pouch cancer), data from index surgery (patient’s age, polyposis phenotype, colorectal cancer in the specimen), upper endoscopic findings based on Spigelman stage for duodenal disease, and medical records concerning the pouch’s evolution: interval from RPC to cancer diagnosis, polyposis severity, previous advanced lesions [≥10 mm/high grade dysplasia (HGD)], length of rectal cuff, number of pouchoscopies and follow-up.

Pouch surveillance in our Colorectal Unit started one year after ileostomy closure, at an endoscopy suite, under conscious sedation. Usually, endoscopic inspection included the full extension of the afferent limb (15–20 cm), the pouch body, and then its lower part (rectal cuff, ATZ, and anastomosis). Any suspicious lesions are biopsied, and polypectomy is performed in those greater than 5 mm and in all lesions situated at the rectal cuff/ATZ area, regardless of size. When conventional endoscopic techniques were not possible, resection was done through a transanal approach.

## RESULTS

During the study period, a total of 192 FAP patients were retrieved from our institutional Registry, seven of whom were excluded according the criteria described before. From the 185 remaining patients, 87 (47.0%) had undergone RPC. During follow-up, three cases of pouch cancer were diagnosed (3.4%). The patients’ demographics and disease features are presented in Tables 1 and 2.

**Table 1:** Demographic and clinical data of familial adenomatous polyposis patients diagnosed with pouch cancer.

Patients	Sex	Age (y) at RPC – pouch cancer	Interval RPC to cancer	FAP type	CRC in specimen	CRC location
Case 1	M	40–53	149 months (12.4 years)	Classical	Yes	Sigmoid and rectum
Case 2	M	34–44	139 months (11.6 years)	Profuse	Yes	Transverse colon
Case 3	M	23–43	240 months (20 years)	Classical	Yes	Right, transverse, sigmoid

RPC: restorative proctocolectomy; FAP: familial adenomatous polyposis; CRC: colorectal cancer; M: Male.

**Table 2:** Risk factors, surveillance and treatment data of pouch cancer patients.

Patients	Previous adenoma treatment	Previous advanced adenoma resected	Local of pouch cancer	Previous upper gastrointestinal tract neoplasia	Treatment & Follow-up
Case 1	No reference	Tubulo-villous adenoma	ATZ (anastomosis)	Papilla cancer	APR (T2N0) 9 years CFSV
Case 2	Yes, 3 resections	25 mm polyp six years after RPC	Rectal cuff	Duodenal adenoma	APR (T1N0) alive 3 years CFSV
Case 3	Yes, 3 resections	Rectal cuff, 15 mm, tubular adenoma	Rectal cuff	Papilla adenoma	ESD T1N0

RPC: restorative proctocolectomy; ATZ: anal transitional zone; APR: abdominoperineal excision; CFSV: cancer-free survival; ESD: endoscopic submucosal dissection.

As seen in Table 2, pouch cancers were diagnosed in three men. Ages varied from 23–40 years at RPC (average: 32.3) and 43–53 years (average: 46.6) at cancer diagnosis. The interval from RPC to pouch cancer diagnosis varied from 11.6 to 20 years (average 14.6 years). All patients presented a CRC in the specimen from the index surgery, two of them with multicenter lesions.

A classical FAP phenotype was found in two patients, while one exhibited a profuse polyposis. During surveillance pouchoscopies, all patients had been diagnosed with advanced adenomas (tubulo-villous histology or size greater than 10 mm) at the rectal cuff area at some moment.

During follow-up, a total of nine pouch endoscopic examinations were performed for these three patients (three examinations each) during the study period. The treatment and follow-up of these cases are presented in Table 2.

**Figure 1:**
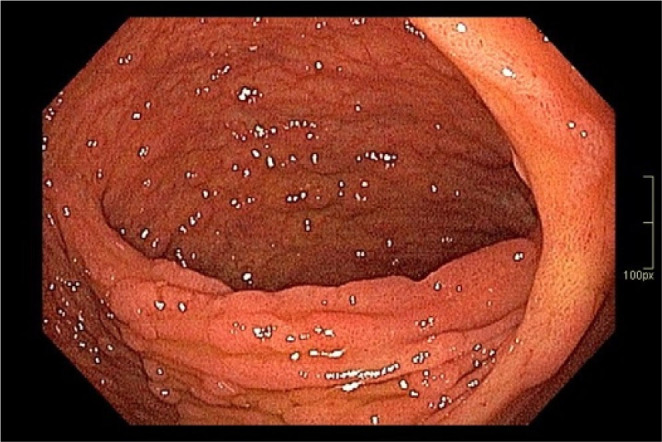
Multiples small polyps at ileal pouch.

**Figure 2:**
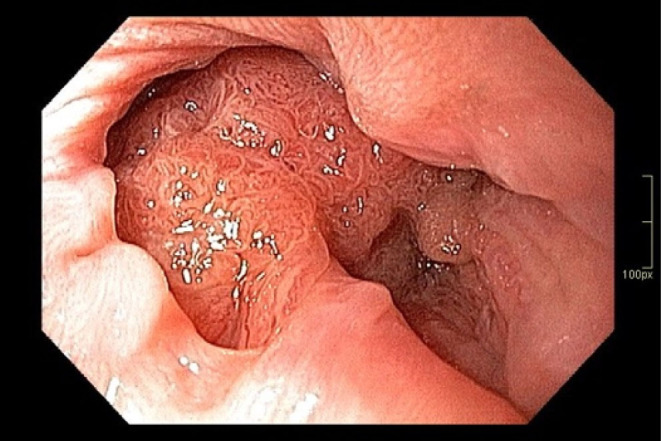
Anal portion of the lesion in the anal transitional zone.

**Figure 3:**
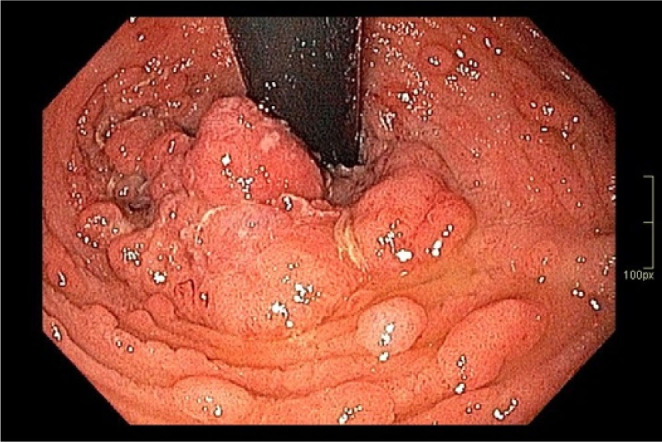
Oral portion of the lesion (on endoscopic retroflex view). The anastomotic line can be seen on the right side.

**Figure 4:**
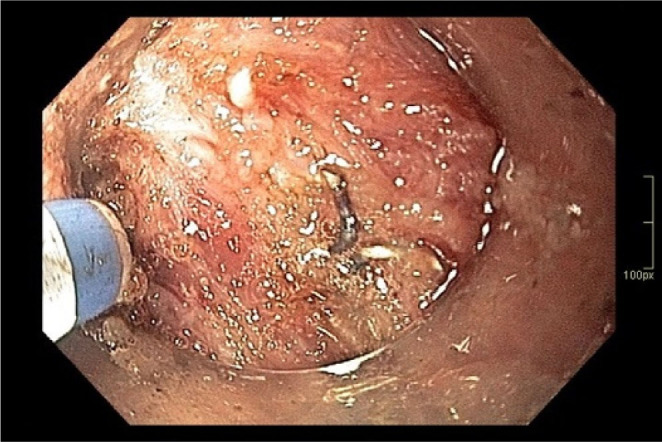
Severe fibrosis and surgical staples.

**Figure 5:**
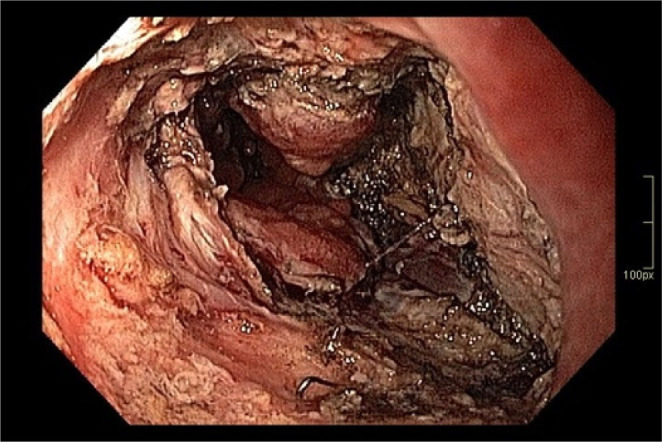
Post-endoscopic submucosal dissection ulcer.

In patient 3, endoscopic examination revealed small adenomatous polyps within the ileal pouch (Figure 1), as well as a 2 cm polypoid lesion exhibiting circumferential involvement of the rectal transitional zone, with extension to the ileal pouch and the anastomotic line (Figures 2 and 3). Histopathological analysis of biopsy specimens confirmed adenocarcinoma. After a multidisciplinary team discussion, the patient was referred for endoscopic submucosal dissection (ESD). During the procedure, severe fibrosis was found at the ileoanal anastomosis, where multiple staples were identified and subsequently removed (Figure 4). Although technically difficult, the lesion was successfully removed (Figure 5).

## DISCUSSION

FAP management raises important controversies regarding issues such as the most appropriate timing for surgery, the preferred abdominal approach, rectal dissection technique (TME or mesenteric dissection) and anastomosis type, among many^30^. In this setting, the choice of RPC turned to be preferred procedure for patients with classical FAP, as it includes rectal resection and provides a better long-term disease control. Common indications criteria include the presence of more than 20 rectal adenomas or large lesions exhibiting high grade dysplasia^10^.

Literature data about pouch neoplastic changes have documented significant differences between series^20,27,37,38,52,53^. As seen in Table 3, data concerning adenoma incidence at the rectal cuff are more limited (13 to 51%), while pouch body adenomas are more frequently found. The rates and severity of pouches neoplasia depend on many issues, such as follow-up time after RPC, length of rectal cuff/ATZ area, previous mucosectomy, the quality of endoscopy (including equipment and endoscopist experience), patients’ phenotype, frequency of previous rectal clearances and follow-up^27,28^.

**Table 3:** Incidence of pouch adenomas according to length of follow-up after restorative proctocolectomy in the literature.

Authors	Location	5 years (%)	10 years (%)	15–20 years (%)
Parc et al.^34^	Paris, France	7	35	75
Le Cosquer et al.^26^	Cleveland, USA	5.9	21.7	40
Tajika et al.^47^	Nagoya, Japan	32	52	68

Table 4 summarizes the overall incidence of adenomas in ileal pouch, ATZ and rectal cuff mucosa in FAP patients, showing a significant variation between literature series. This wide range (13 to 76%) is partly related to different disease features, length of follow-up, quality of endoscopic surveillance, a more aggressive type of polyposis in patients selected for RPC, anastomosis technique and others.

**Table 4:** Overall adenoma risk in familial adenomatous polyposis literature series.

Author	Period	Origin	FAP	Follow-up (years)	Adenoma risk (%)
Friederich et al.^14^	Cohort	Nijmegen, Holland	212	7.9	45.0
Tonelli et al.^49^	1992–2012	Florence, Italy	69	10	64.9
Pommaret et al.^39^	2003–2008	Paris, France	118	15	48.3
Boostrom et al.^5^	1972–2007	Rochester, USA	117	15	25.6
Ganschow et al.^17^	Cohort	Munich, Germany	192	12.8	46.9
Patel et al.^37^	1997–2018	London, St. Mark’s	249	10	76.0
Aelvoet et al.^1^	2001–2011	Amsterdam, Holland	111	20	72.0
Lee et al.^28^	1983–2019	Ohio, USA	165	10.1	28.5
van Duijvendijk et al.^50^	1981–1997	Multicenter*	97	5.5	13.4
von Roon et al.^51 †^	1977–2003	London, St. Mark’s*	189	10.3	19.0–38.0 %
von Roon et al.^52 †^	1978–2007	London, St. Mark’s*	206	10.3	22.6–51.1 %
Ozdemir et al.^33 †^	1983–2010	Ohio, USA*	260	9.2	20.9–33.9%

FAP: Familial Adenomatous Polyposis.

* cuff/ATZ adenoma rate;

† polyp incidence after mucosectomy and stapled anastomosis.

In 2013, Tajika et al.^46^ reviewed 25 reports and found that the incidence of adenomas in the ileal pouch varied from 6.7 to 73.9%. More recently, Gavric et al.^18^ published a systematic literature review of 54 papers to assess long-term rates of neoplasia development after prophylactic surgery in 5,010 FAP patients. They found a 9.4–85% adenoma rate and a 12% risk for advanced adenoma ten years after IPAA.

It is widely recognized that the incidence of pouch polyps increases with pouch age (Table 3). Other studies indicated incidences of 7–16%, 35–42%, 75% and 45–78% at five, ten, 15 and 20 years, respectively^5,17,46^. While most patients (85%) are expected to be free of pouch adenomas five years after IPAA, this scenario will change to a 72–85% risk at 20 years of follow-up^17,46^.

The present series represents the experience of our Colorectal Unit in the management of FAP patients undergoing RPC at a public university hospital. Over four decades, we diagnosed three cases of pouch cancers in a group of 87 RPC (3.4%). The first of these cases had been previously reported in a previous publication as the 15^th^ documented case of pouch cancer in the literature at that time^8^.

Since pouch cancer is a rare event even in specialized centers, its true incidence is very difficult to estimate. Table 5 presents a list of cases published in series of RPC from important centers, with rates varying from 0.8 to 3.4%. In series with 69–260 FAP, 29 pouch cancers were described (one to five patients in each series). In a theoretical supposition that these 29 patients were diagnosed in the group of 1,423 FAP patients listed in Table 5, we would reach a median pouch cancer incidence of around 2.0%.

**Table 5:** Incidences of pouch cancer in familial adenomatous polyposis series from different countries.

Author	Study origin	RPC for FAP	Age at pouch cancer (years)	Cancer rate n (%)	Interval to cancer (years)
Friederich et al.^14^	Dutch FAP Registry	212	32,35,36,37	4 (1.8 %)	6 to 14
Pommaret et al.^39^	Poznan, Poland	165	-	5 (3.0 %)	11 to 20
Tonelli et al.^49^	Florence, Italy	69	29, 59	2 (2.9 %)	3, 11
Boostrom et al.^5^	Rochester, USA	117	36	1 (0.8 %)	23.6
Ozdemir et al. ^33^	Cleveland, USA	260	-	4 (1.5%)	8 to 11
Wasmuth et al.^53^	Norway FAP Registry	61	-	1 (1.6%)	-
Pasquer et al.^36^	Lyon, France	92	30	1 (1.1%)	-
Patel et al.^38^	London, UK	249	-	5 (2.0%)	16.5
Aelvoet et al.^1^ *	Amsterdam. Holland	111	-	3 (2.7%)	12.6
Campos et al., 2025	São Paulo, Brazil	87	43,45,62	3 (3.4%)	11 to 20

RPC: restorative proctocolectomy; FAP: familial adenomatous polyposis.

* European FAP Consortium.

Table 5 did not include 21 pouch cancers published as case reports. Similarly, we did not include another eight patients previously included in series pertaining to institutions that are already listed in this table^28,32,40,51,52^ and three others reported in multicenter reports^6^. According to these criteria, there are approximately 60 pouch cancer cases reported in the literature so far.

Recently, two large series focused on pouch cancer incidence. Bouchiba et al.^6^ published an international multicenter cohort study (1990–2023) with results from the European FAP consortium. They found only three cases of pouch cancer among 319 FAP (0.9%) and attributed the small number of cases found in the last decades to improved surgical selection and close endoscopic surveillance. In another important review of 54 papers (1946–2023) including 5,010 FAP, Gavric et al.^18^ found a 9.4–85% pouch adenoma rate and 45 pouch cancers detected in 2.3 to 33 years after RPC.

It is important to note that our own incidence rate of 3.4% seems to be one of the highest among other studies. However, seven RPC patients were excluded due to follow-up smaller than three years (4), other forms of pouch (1) and manual pouch-anal anastomosis following mucosectomy (2). Then, thinking that the eventual chance of this group to develop pouch cancer would still be small, we could admit a rate of three cases among 94 patients (3.1%), much closer to other reported rates^1,3,49^.

We should also consider that our hospital is a public tertiary referral center that receives patients with a relatively more severe polyposis, mainly because we work in a specialized ambulatory designated to treat FAP patients. For us, it is relatively common to manage patients older than 30 years of age or with FAP-associated CRC, as reported in previous publications^9,13^.

Risk factors for cancer development have been discussed, such as male sex^17^. Controversial issues include severe colonic polyposis (more than 1,000), CRC in the index specimen, and age at surgery (>50 years)^22,28,45,49^. In the present series, our patients displayed some adverse features, such as male sex, profuse polyposis, CRC in the specimen, multiple cancers, previous advanced adenoma, and an association with upper digestive adenomas (Tables 2 and 4).

In the literature, many series reported a long interval between RPC and cancer development, similarly to our three patients (3–20 years). In addition, it has been recognized that pouch adenomas prior to ten years after RPC may be associated with the subsequent development of advanced adenomas greater than 10 mm, and those type of lesions usually precede tumors with HGD^38,47,49^.

While body cancers arise from the ileal tissue, cuff neoplasia are formed at the residual rectal mucosa at the ATZ or from the ileoanal anastomosis^24^. These cuff tumors have been detected in 0.5–3.6% of patients^27,43,52^, and pouch body tumors are considered rare^43,46^. In a retrospective review of 249 FAP cases from the St. Mark’s Hospital Polyposis Registry, Patel et al.^38^ found five pouch cancers (0.2%), four in the cuff and one in the body. Adversely, pouch body and cuff/anastomosis tumors were found in 30 and 15 patients, respectively, in the largest review of the literature so far^18^. This data demonstrates that pouch carcinogenesis mechanisms have yet to be better understood^24,26^.

One common observation is that pouch adenoma incidence is approximately 2–3 folds higher among stapled anastomosis patients when compared to handsewn IPAA^27,50,52^. In a large review, incidence varies from 33.9–57% and 0–33% in stapled and handsewn anastomosis, respectively^18^. Conversely, the difference in the incidence of pouch cancer detected after both anastomosis appears to be relatively modest (Table 6).

**Table 6:** Incidences of adenoma and cancer at the anal transitional zone after mucosectomy with handsewn or double-stapled ileal-pouch anastomosis.

Authors	FAP	Adenoma rates after hand-sewn and stapled anastomosis (%)	Cancer rates after hand-sewn and stapled anastomosis (%)	Follow-up (years)
van Duijvendijk et al.^50^	126	10–31	0–0	5.5
Remzi et al.^40^	119	14–28	0–1.3	5.8–3.6
von Roon et al.^51^	140	19–8%	2–0	10.3
von Roon et al.^52^	206	22.6–51.1	1.3–0	10.3
Ozdemir et al. ^33^	260	20.9–33.9	1.2–1.7	12.9–7.9

FAP: familial adenomatous polyposis

An expanded analysis of the literature regarding this topic was provided by Ozdemir et al.^33^, who showed that although the adenoma risk at the ATZ was lower among mucosectomy patients (21 x 34%, p=0.03), carcinoma risk (1.2 x 1.7%) and time to cancer development (8 x 11.3 years) were not statistically different. These findings reinforce the need for careful surveillance of the ATZ, even though pouch cancers may develop despite rigorous IPAA inspection.

Certainly, although mucosectomy and handsewn IPAA may protect the distal pouch area against adenoma development, this benefit should be balanced with the choice of a technically complex procedure that may lead to higher rates of complications and worse bowel function. For this reason, we have chosen to perform a double-stapled anastomosis in the great majority of our patients, reserving the mucosectomy only for those with carpeted ATZ or low rectal cancer. Technical simplicity and better functional outcomes provided by the stapled option represent the tradeoff to the development of neoplasia in the ATZ area.

Based on the development of modern endoscopic technologies, we have performed polypectomies to treat greater than 5 mm or more advanced lesions in the pouch during follow-up, because we believe that this attitude may lower the risk of pouch excision in the long-term, as shown in previous studies^2^.

As demonstrated herein, the finding of a pouch cancer is a rare event that may even occur even despite adequate surveillance^19^. For this reason, outcomes after treatment have been scarcely reported in the literature. Intervention modalities include endoscopic techniques (ablation, mucosal or ESD), surgical transanal excision, pouch advancement, redo ileoanal anastomosis (technically demanding) or pouch excision. All these choices will be confronted depending on tumor features (grade, location, size, staging), patient’s clinical conditions and medical expertise on endoscopic and surgical techniques^13^. For superficially invasive lesions, ESD may be considered a viable, minimally invasive alternative. This approach allows for local resection, promotes organ preservation and is associated with lower morbidity^31^. In Case 3 of our series, for example, the lesion was successfully managed with local endoscopic resection by ESD, as seen in the corresponding images.

Due to a disruption of anatomical planes during the previous surgery, rectal cuff lesions will usually present as locally advanced lesions. Neoadjuvant therapy is highly controversial. Excision is performed through abdominoperineal or exenterative procedures according to local and distant dissemination. These procedures are associated with significant morbidity.

All the data presented here suggest that RPC is not a definitive solution for FAP, as patients remain at risk of developing adenomas and cancer in the pouch body or rectal cuff/ATZ area. This potential risk is considered greater than previously thought, with increasing incidence and severity of pouch neoplasia with time after RPC, making careful and well-planned surveillance mandatory^28^.

Most patients do not develop an adenoma within five years of pouch formation, and the development of HGD and cancer are rare events. Pouch endoscopy may miss dysplasia in flat invisible lesions, but the progression of pouch adenomas is fortunately slow, and the greatest risk appears to occur after ten years, suggesting that surveillance during this period may be reduced for most patients, provided they have early engagement in the surveillance program^29,44^.

Surveillance frequency has varied from routine (1–2 years) or according to individual findings and symptoms. This depends on the identification of high-risk patients during the first decade of follow-up (uncontrolled pouch polyposis, large lesions or high-grade dysplasia), allowing to stratify patients into high and normal risk groups for the future development of advanced lesions. Consequently, the adoption of a personalized surveillance strategy and the extension of intervals in the low-risk group may add effectiveness and optimize economic resources^37,48^.

Our study has some limitations. Despite being the largest Brazilian cohort to date, pouch cancer is a rare event that occurs in a disease that is also not so common. Thus, the numbers are usually small. Moreover, endoscopic surveillance during the last four decades has changed a lot in terms of frequency and quality, influencing adenoma detection rate and treatment. Moreover, patients do not accept annual pouchoscopies so easily.

## CONCLUSIONS

Pouch cancer is a rare disease diagnosed in incidences varying from 0.8 to 3.4% in worldwide FAP series. Male patients, presence of CRC in the RPC specimen, colorectal phenotype, and association with duodenal adenomas are considered the main risk factors. Pouch adenomas develop after both handsewn or stapled anastomosis. Pouch polypectomy might prevent the development of adenocarcinomas, as patients under surveillance are diagnosed with more localized disease.
